# Bidirectional promoters in *Escherichia coli*: regulatory rules and implications for gene expression noise

**DOI:** 10.1093/nar/gkag028

**Published:** 2026-01-22

**Authors:** Emily A Warman, David Forrest, Anne M Stringer, Joseph T Wade, David C Grainger

**Affiliations:** School of Biosciences, University of Birmingham, Edgbaston, Birmingham B15 2TT, United Kingdom; School of Biosciences, University of Birmingham, Edgbaston, Birmingham B15 2TT, United Kingdom; Wadsworth Center, New York State Department of Health, Albany, NY 12201, United States; Wadsworth Center, New York State Department of Health, Albany, NY 12201, United States; Department of Biomedical Sciences, School of Public Health, University at Albany, Albany, NY 12222, United States; School of Biosciences, University of Birmingham, Edgbaston, Birmingham B15 2TT, United Kingdom

## Abstract

In prokaryotes, bidirectional promoters are pseudo-symmetrical DNA sequences that stimulate divergent transcription. Ubiquitous, and far more likely to drive messenger RNA production than directional promoters, nothing is known about their control. For example, symmetry allows bidirectional promoters to engage RNA polymerase in two possible orientations. As one binding event prevents the other, there is potential for regulation at this step. Here, we show that basal transcription, from all five tested bidirectional promoters, is too low for RNA polymerase competition. Hence, synthesis of one RNA does not impact the divergent pair. Conversely, if transcription in one direction is substantially activated, divergent RNA production can be repressed. Often, this results from RNA polymerase competition alone. Unexpectedly, this also impacts population-level gene expression noise. Specifically, if transcription is constrained, by RNA polymerase interference, cell-to-cell variation is reduced. We anticipate that our findings will help to establish rules for understanding bidirectional promoters, which have hardly been studied, in many bacteria.

## Graphical Abstract

**Graphical Abstract:**
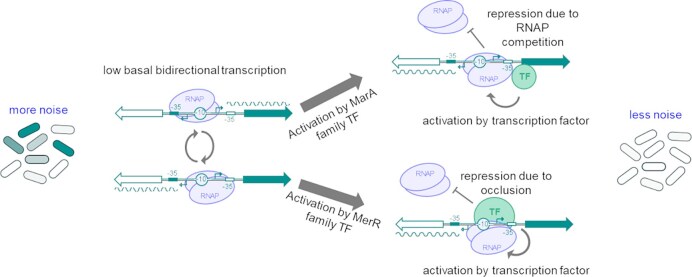

## Introduction

In bacteria, transcription requires initial binding of RNA polymerase to a DNA sequence called a promoter [1]. A promoter is a short double-stranded DNA segment, essential for the specific initiation of RNA production from a defined location [1]. The core housekeeping promoter consists of two hexameric elements, at positions -10 and -35, with respect to the transcription start site (TSS, or +1). Both sequences have a well-defined DNA consensus, 5′-TATAAT-3′ and 5′-TTGACA-3′, respectively, and interact with the same RNA polymerase subunit [2]. In *Escherichia coli*, this subunit is called σ^70^ [2]. The -10 motif binds σ^70^ domain 2 and is unwound during transcription initiation [3]. Conversely, the -35 hexamer remains double stranded and captures σ^70^ domain 4 [4]. A gap of 17 bp between the core promoter elements is optimal [5]. Within this spacer region, AT-rich sequences around position -18 are stimulatory, and compensate for a missing -35 hexamer [6, 7]. However, the -10 element is largely indispensable due to its role in DNA opening. Ancillary promoter elements can increase RNA polymerase binding but are not always present [1, 8].

Many promoter sequences are poor and rely on activator proteins to stimulate transcription [2]. Such activators may bind DNA, and then capture the transcriptional apparatus, or bind RNA polymerase independently before guiding it to a promoter. These pathways, described as recruitment and pre-recruitment, respectively, both require activator–RNA polymerase contacts [9–11]. While common, such contacts are not always the primary mechanism of activation [12]. For example, MerR family activators bind between incorrectly spaced -10 and -35 hexamers. As a result, the DNA kinks and promoter elements are brought into correct alignment [12, 13]. Repressor proteins also target the core promoter and sterically block RNA polymerase recognition [2]. Indeed, many regulators may be activators or repressors, depending on their binding location [14, 15].

Previously, we showed that inherent -10 element symmetry allows divergent transcription from many promoters [16]. For example, in *E. coli*, 19% of all RNAs detected were generated in this way [16]. In such cases, TSSs, on opposite DNA strands, occur either side of, or within, a shared -10 element. Hence, a short promoter DNA sequence can be sufficient for transcription in both directions (Supplementary Fig. S1). We refer to these promoters as bidirectional and additional promoter elements, or regulator binding sites, can influence the level of transcription in a specific direction [16]. The most common distance separating divergent TSSs at bidirectional promoters is 18 bp, with distances of 10 and 23 bp also common (Supplementary Fig. S1). While these distances are preferred, small variations are possible. This is because the distance between the -10 element and TSS is not always the optimal 7 bp [16]. Hence, while -10 element configurations remain as shown in Supplementary Fig. S1, adjacent TSS positions may differ slightly. Found in all prokaryotes, bidirectional promoters are most frequently associated with messenger RNA (mRNA) synthesis [16, 17]. Hence, these promoters must have a major role in controlling gene expression. Importantly, steric constraints prevent simultaneous binding of opposing RNA polymerase molecules to bidirectional promoters [18]. It seems likely such interference is exploited for regulatory purposes [16]. Note that such direct clashes, at the site of RNA polymerase binding, do not impact separate promoters that are divergent, which we do not categorize as bidirectional.

In this work, we show that all bidirectional promoters tested have low basal activity levels. This minimizes interference caused by RNA polymerase competition. As a result, loss of transcription, in one direction, does not substantially aid production of the divergent RNA. Conversely, competition may arise if transcription increases; opposing RNA synthesis can be constrained. Consistent with this, activators, which enhance transcription in one orientation, tend to repress the divergent RNA. Most often, this results from RNA polymerase competition alone. Hence, if uncoupled from activation, regulator binding has no such effect. As well as changing the level of transcription, RNA polymerase interference impacts gene expression noise, defined as cell-to-cell variation [19]. Thus, if transcription in one direction is hindered, resulting gene expression is less variable. By virtue of their unusual properties, MerR family regulators induce different behaviour at bidirectional promoters. Specifically, these factors sterically restrict RNA polymerase to a single binding orientation. As a result, and although interference does not occur, transcription in one direction is always blocked.

## Materials and methods

### Strains and plasmid cloning

Bacterial strains, plasmids, and oligonucleotides are listed in Supplementary Table S1. Promoter DNA was amplified using the genome as a polymerase chain reaction (PCR) template and inserted within plasmid pLSR by HiFi Assembly (NEB) or digestion/ligation. Mutations in promoters were introduced by oligonucleotides during PCR. Plasmids were purified with ZymoPURE II Plasmid Midiprep kits (Zymo Research). To constitutively express regulators in cells prior to LacZ assays, *soxS* was cloned in plasmid vector pJ203 (Atum) downstream of a promoter with low-level constitutive activity. Plasmids for protein overexpression were generated by cloning *soxR* or *acrR* in pET28a.

### Protein purification

MarA and SoxS were purified as described previously using a His-tag and Nickel column chromatography [20]. AcrR and SoxR were purified using the methods described by Li *et al.* [21] or Chandler and Demple, respectively [22]. Briefly, plasmids were used to transform T7 Express cells (NEB) and protein overexpression was induced by 1mM IPTG. Cells were lysed with a tip sonicator and the lysate was applied to a His-trap chromatography column (Cytivia). Proteins were eluted from the column with imidazole and stored at −20°C. Cyclic AMP receptor (CRP) was purified using cAMP-agarose as described previously [23].

### *In vitro* transcription assays

To understand the basal properties of bidirectional promoters, we selected DNA sequences that generated divergent RNAs in genome-wide TSS mapping experiments [24] and in our *in vitro* transcription assays. For the latter experiments, reactions contained 300 ng plasmid DNA, 40 mM Tris Acetate, 10 mM MgCl_2_, 100 mM KCl, 200 μM ATP/CTP/GTP, 10 μM UTP, and 2 μCi [α-^32^P] UTP (Hartmann). Reactions with CRP also included 0.2 mM cAMP. Proteins were added where appropriate in a concentration gradient: 0.25–3 μM for MarA/SoxS, 0.4–4 μM for CRP, 0.05–0.4 μM for SoxR, and 0.5–4 μM for AcrR. Reactions were started with the addition of one unit of RNA polymerase σ^70^ holoenzyme (NEB). This is sufficient to incorporate 1 nmol NTP into RNA in 10 min at 37°C. After 10-min incubation at 37°C, reactions were stopped with an equal volume of formamide containing 100 mM ethylenediaminetetraacetic acid. Transcripts were separated by denaturing polyacrylamide gel electrophoresis on 8% (w/v) gels (Protogel). After drying, gels were exposed to a phosphoscreen overnight and visualized using BioRad Image Lab software. For band intensity analysis, band volumes for each transcript were adjusted for background intensity and normalized using the intensity of the RNAI internal control. Where multiple transcripts initiated from the same promoter, in the same direction, band volumes were added.

### Electrophoretic mobility shift assays

Fragments were generated using PCR, purified by gel extraction, and radiolabelled with [γ‐^32^P]‐ATP (Hartmann) and PNK (NEB). The DNA was incubated at 37°C with a concentration gradient of purified protein in the presence of 40 mM Tris Acetate, 10 mM MgCl_2_, 100 mM KCl, 25 ng/μl Herring Sperm DNA and 7.5% (v/v) glycerol. DNA–protein complexes were separated from free DNA on a 5% (w/v) non-denaturing polyacrylamide gel (National Diagnostics). Gels were exposed to a phosphoscreen overnight and visualized using BioRad Image Lab software.

### Genome engineering

Changes were made to the P*acrA/acrR* region in *E. coli* JCB387 using an adapted Gene Doctoring protocol [25]. First, the *acrR* gene was swapped for a kanamycin resistance cassette by λ red recombination. A mutation in the *acrR* -35 element was included in the homologous region, upstream of the kanamycin resistance cassette, so replaced the wild-type chromosomal sequence during integration. The cassette was then removed using the flip recombinase. The PAmCherry encoding gene, and an adjacent Kan cassette, was then inserted between *acrA* and *acrB*. DNA changes were confirmed using PCR and Sanger sequencing.

### β-galactosidase assays

Promoter sequences were inserted upstream of *lacZ* in plasmid pRW50. Activity of β-galactosidase was measured in cell lysates as a reporter of promoter activity. Assays were done in triplicate following the Miller protocol [26]. Data shown are mean values and error bars indicate standard deviation.

### Microscopy

Single colonies were used to inoculate M9 minimal media +0.3% glucose and incubated at 37°C overnight. These were diluted into fresh media and grown to an OD_650_ of 0.3–0.6. Cells from 1.5 ml of culture were collected by centrifugation and resuspended in 10 μl of fresh media. This was pipetted onto a 1% agar pad, on a microscope slide, and covered with a cover slip. Images were captured using a Zeiss LSM 780 confocal microscope, under simultaneous excitation with a low-intensity 405-nm laser (to photoactivate PAmCherry) and a high-intensity 561-nm laser. Fluorescence was measured with the Fiji package for ImageJ.

## Results

### The basal activity of bidirectional promoters minimises RNA polymerase competition

**Figure 1.**
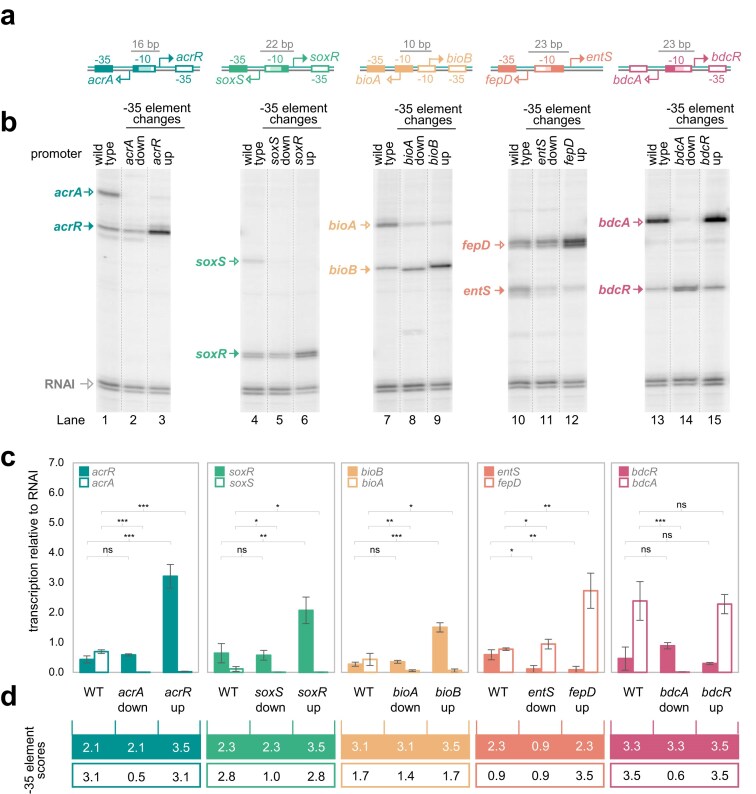
Basal transcription from bidirectional promoters is too low for substantial competition between RNA polymerase molecules. (**a**) Bidirectional promoter regions used in this study. Schematic representation of the bidirectional promoter regions used in this study. TSS locations are those previously mapped using cappable-seq [32] and are indicated by bent arrows. Distances between divergent TSSs are shown above each schematic. Promoter -10 and -35 elements are shown as boxes and labelled. Filled and open boxes indicate elements used for forward and reverse transcription, respectively. Lighter shading highlights regions of the -10 element shared during forward and reverse transcription. (**b**) Transcription from bidirectional promoter regions and their derivatives *in vitro*. The panel shows results of *in vitro* transcription assays for each bidirectional promoter region and derivatives with -35 element mutations. In each case, the -35 element changes are designed to impede (denoted ‘down’) or enhance (denoted ‘up’) transcription in the indicated direction (see labels above each lane). One unit of RNA polymerase holoenzyme (NEB) was used per reaction. The RNAI transcript is generated from the plasmid DNA replication origin and serves as an internal control. (c) Quantification of forward and reverse transcription from each DNA template. Band intensities were measured and used to determine the amount of each divergent transcript relative to the RNAI control. A two-tailed paired Student’s *t*-test was used to calculate *P*, where * <.05, ** <.01, and *** <.001. Error bars show standard deviation from at least three independent replicates.(**d**) Promoter -35 element scores for forward and reverse transcription. For each DNA template, -35 motif scores are indicated for forward (filled boxes) or reverse (open boxes) transcription. Scores were calculated using a position weight matrix [31]; the consensus -35 element has a score of 3.5. The colour code matches that used in panel (c).

Our first goal was to understand whether bidirectional promoters have sufficient basal activity, resulting from the DNA sequence alone, to induce RNA polymerase competition. Note that, in *E. coli*, promoter activity is limited by both RNA polymerase recruitment and subsequent promoter escape [27–29]. Hence, unless promoters have a perfect match to the various consensus elements, and bind RNA polymerase unusually tightly, promoter activity and promoter occupancy positively correlate [28, 29]. Previously, we found that 24% of bidirectional promoters, in *E. coli*, are between divergent genes and control production of two mRNAs [16]. Often the transcripts encode functionally linked proteins [30]. Since these are particularly interesting examples to study, we selected five such regions: between divergent gene pairs *acrA*/*acrR, soxS*/*soxR, bioA*/*bioB, fepD*/*entS*, and *bdcA*/*bdcR*. In each case, the divergent TSSs are separated by between 10 and 23 base pairs (shown schematically in Fig. 1a). Each bidirectional promoter sequence was cloned, between transcriptional terminators, in plasmid pLSR. The resulting constructs were then used as templates for *in vitro* transcription. Note that because each terminator is a different distance from the cloned DNA, the divergent RNAs produced are different sizes. The results are shown in Fig. 1b (lanes 1, 4, 7, 10, and 13). As expected, two RNAs, resulting from divergent transcription, were detected in each case. Levels of forward and reverse transcription are quantified in Fig. 1c (see wild-type variants). The figure also shows position weight matrix scores for each -35 hexamer, where a maximum score of 3.5 indicates a consensus sequence (Fig. 1d) [31].

Logically, if RNA polymerase molecules compete for binding, reduced transcription in one orientation should permit more in the other. To test this, we mutated one of the two -35 hexamers either side of each -10 element (decreasing the -35 element scores in Fig. 1d). The precise mutations are shown in Supplementary Table S2 and results are Fig. 1b (lanes 2, 5, 8, 11, and 14). For each reaction, the RNA impacted by mutation is indicated above the lane. While transcription in the expected direction was reduced, there was little impact on divergent RNA production. The changes in forward and reverse transcription are quantified in Fig. 1c (compare wild-type and ‘down’ variants). We conclude that basal transcription, from the bidirectional promoters tested, generates little competition between RNA polymerase molecules. Note that in this context, we refer to only steric competition, where RNA polymerase binding in one orientation blocks the other. We do not refer to competition between promoters for the RNA polymerase pool, since this is not limiting in our conditions. Hence, increasing or decreasing the concentration of RNA polymerase had no effect on ratio between forward and reverse transcription for any bidirectional promoter tested (Supplementary Fig. S2, ‘wild-type’ lanes). Similarly, the ‘down’ promoter mutations had the same impact across a range of RNA polymerase concentrations (Supplementary Fig. S2, ‘down’ lanes).

### RNA polymerase competition arises if bidirectional promoter activity increases

While marked RNA polymerase competition was not apparent in initial experiments, we reasoned increased transcription, in one direction, might generate such effects. To test this, we introduced mutations that improved one -35 element in each pair (Supplementary Table S2, ‘up’ mutations). Transcripts generated are shown in Fig. 1b (lanes 3, 6, 9, 12, and 15) and -35 element scores are in Fig. 1d. Except for *bdcR*, the sequence changes stimulated transcription in the expected direction. Furthermore, transcription in the opposite direction was greatly reduced. The results are quantified in Fig. 1c (compare wild-type and ‘up’ variants). We conclude that substantial RNA polymerase competition can arise at bidirectional promoters, but only if levels of transcription are sufficiently high.

### Activation by pre-recruitment can stimulate competition between RNA polymerase molecules at bidirectional promoters

**Figure 2.**
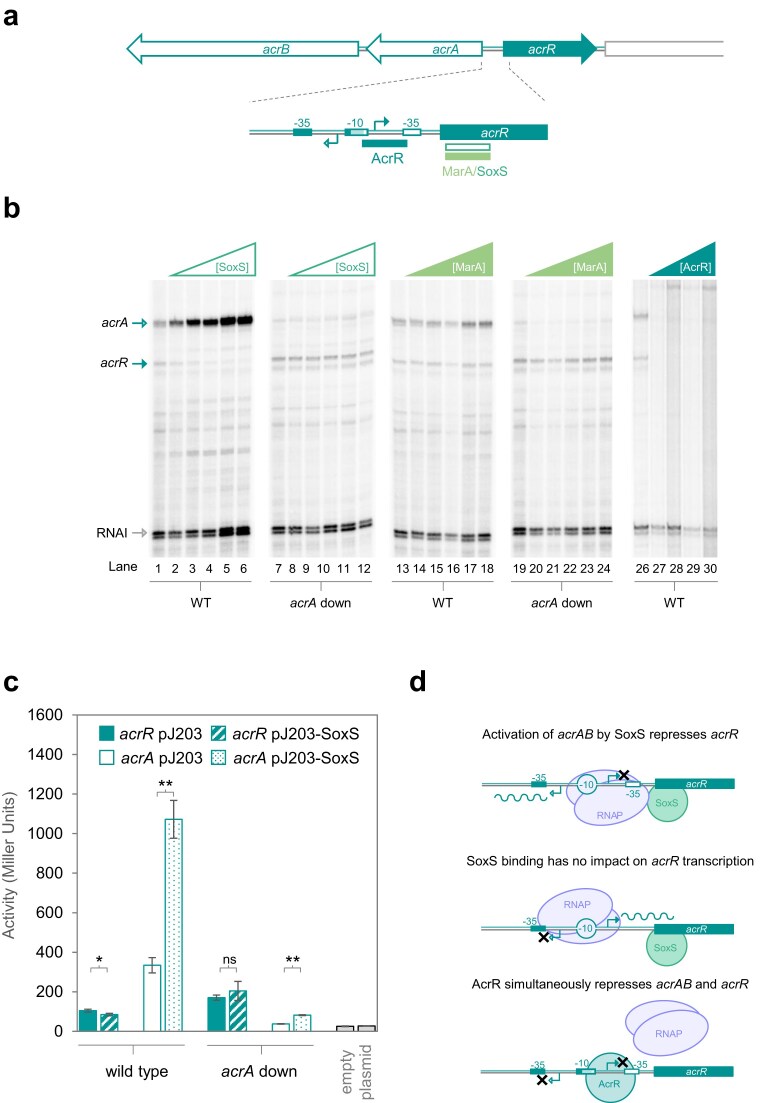
SoxS induces competition between RNA polymerase molecules at the bidirectional promoter P*acrA/acrR*. (**a**) The *acrAB/acrR* locus. A schematic showing organization of the *acrAB* operon and divergent *acrR* gene. Each open reading frame is a block arrow. The expansion shows organization of the bidirectional promoter P*acrA/acrR*. TSSs are shown as bent arrows. Promoter elements for transcription in the direction of *acrR* and *acrA* are filled and open boxes, respectively. The shared portion of the -10 hexamer, used for both forward and reverse transcription, has lighter shading. Extended boxes indicate binding sites for gene regulatory proteins. The closely related factors SoxS and MarA recognise exactly the same sequence and activate transcription in the direction of *acrA*. The AcrR protein is a repressor. (**b**) Transcription from P*acrA*/*acrR* and derivatives *in vitro*. The panel shows results of *in vitro* transcription assays for each bidirectional promoter region and derivatives with -35 element mutations. In each case, the -35 element changes are designed to impede (denoted ‘down’) transcription in the indicated direction (see labels below each lane). The RNAI transcript is generated from plasmid DNA replication origin and serves as an internal control. Two units of RNA polymerase holoenzyme (NEB) were used per reaction. Where present, SoxS and MarA were used at concentrations of 0, 0.25, 0.5, 1, 2, or 3 μM. (**c**) Transcription from P*acrA*/*acrR* and derivatives *in vivo*. The bar chart shows results of β-galactosidase assays. Experiments were done in triplicate, and error bars show standard deviation. The bidirectional promoter region was cloned upstream of *lacZ* either in the forward orientation (to monitor *acrR* transcription, solid bars) or in the opposite orientation (to detect expression of *acrA*, open bars). Bars labelled ‘empty plasmid’ show values for promoterless *lacZ* in each strain. SoxS was expressed ectopically, from a low-level constitutive promoter in pJ203-SoxS, as indicated. A two-tailed paired Student’s *t*-test was used to calculate *P*, where * <.05 and ** <.01. (**d**) Models for control of P*acrA*/*acrR* by SoxS and AcrR. Proteins are shown as ovals and transcripts as wavy lines. Crosses indicate loss of transcription in a given direction.

Our data suggest that the basal activity of many bidirectional promoters is close to, or below, the level needed for significant interference between RNA polymerase molecules (Fig. 1). We next sought to understand whether transcriptional activators might stimulate competition. To do this, we turned our attention to the P*acrA*/*acrR* bidirectional promoter, located between *acrR* and the *acrAB* operon (Fig. 2a). The *acrR* gene encodes a TetR family auto-repressor and *acrAB* encodes components of the AcrAB/TolC efflux system [30, 33–35]. Expression in the *acrAB* direction can be activated by SoxS, or a closely related transcription factor called MarA [36]. These proteins recognize the same DNA site and, while they can bind this target independently *in vitro*, stimulate transcription by pre-recruitment of RNA polymerase [10, 11, 32]. Results of *in vitro* transcription assays, with P*acrA*/*acrR*, in the presence of SoxS or MarA, are shown in Fig. 2b. Lanes 1–6 indicate strong activation of transcription in the *acrA* direction by SoxS. Simultaneously, *acrR* is completely repressed. While this likely results from increased RNA polymerase competition, the location of DNA-bound SoxS could hinder elongation of the *acrR* transcript (Fig. 2a). To distinguish between these possibilities, we used the ‘*acrA* down’ variant described above, with a deleterious -35 element mutation. As expected, this promoter derivative bound SoxS with the same affinity as the wild-type promoter (Supplementary Fig. S3). Conversely, activation by SoxS was abolished (Fig. 2b, lanes 7–12). Hence, the mutation in the -35 element has uncoupled SoxS binding from activation of *acrA* transcription. Strikingly, in this situation, transcription in the direction of *acrR* was no longer repressed. Hence, interference between RNA polymerase molecules, rather than SoxS binding *per se*, must be responsible. Similar effects are apparent *in vivo* using promoter::*lacZ* fusions that detect transcription in either the *acrA* or *acrR* direction (Fig. 2c). Briefly, SoxS stimulates transcription of *acrA* (compare open and speckled bars for the wild type promoter). This simultaneously reduced transcription of *acrR* (compare equivalent filled and striped bars). The ‘*acrA* down’ mutation prevents all such effects.

Interestingly, MarA activates transcription in the direction of *acrA* much less efficiently than SoxS. Accordingly, transcription in the direction of *acrR* is unaffected (Fig. 2b, lanes 13–18). This is likely due to insufficient competition between RNA polymerase molecules. As expected, AcrR represses production of both RNAs (lanes 26–30). Our models for control of divergent mRNA production are shown in Fig. 2d. We conclude (i) that sufficient activation by pre-recruitment can repress divergent transcription at bidirectional promoters, (ii) that this is a consequence of increased competition between RNA polymerase molecules, and (iii) that DNA-bound SoxS does not hinder RNA elongation. The regulatory logic is intriguing and resembles a positive feedback loop; increased *acrA* mRNA synthesis reduces transcription of *acrR*, encoding a repressor of both genes.

### Activation by recruitment can stimulate competition between RNA polymerase molecules at bidirectional promoters

**Figure 3.**
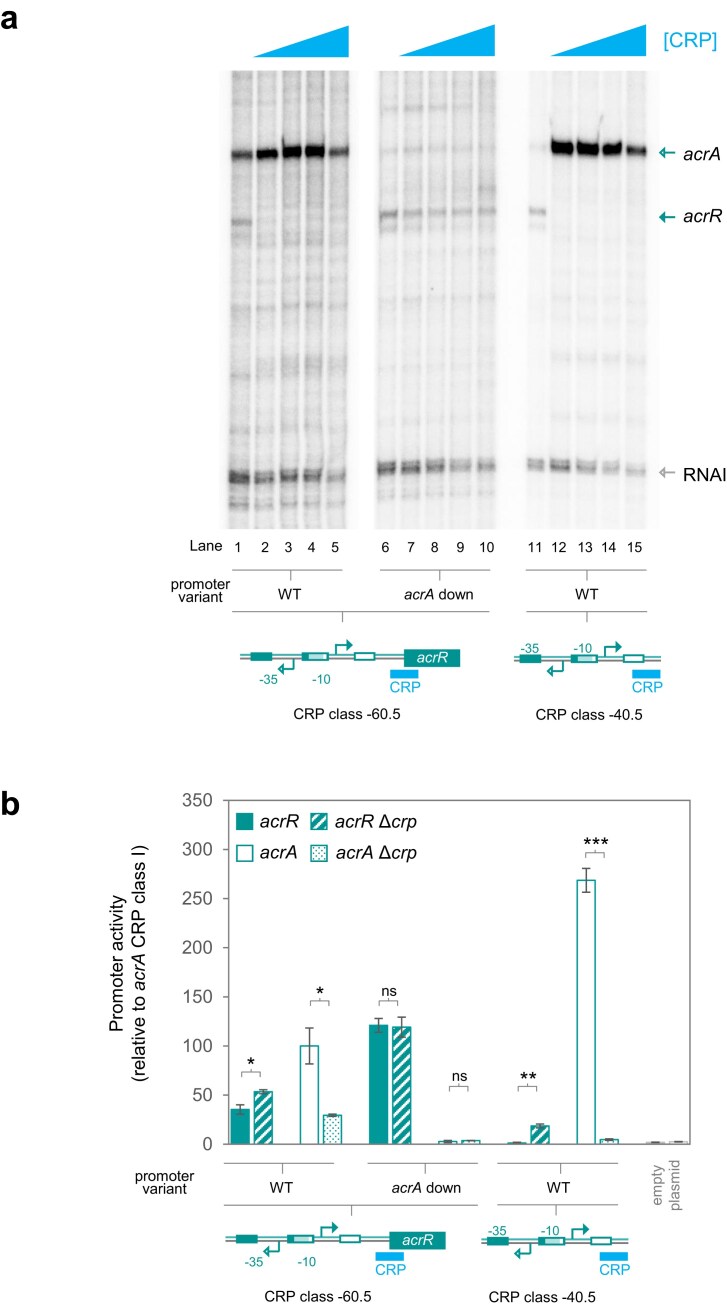
Competition between RNA polymerase molecules at semi-synthetic P*acrA/acrR* derivatives dependent on CRP. (**a**) Transcription from CRP regulated P*acrA*/*acrR* and derivatives *in vitro*. The panel shows results of *in vitro* transcription assays for each bidirectional promoter region and a derivative with -35 element mutations. The -35 element changes are designed to impede (denoted ‘down’) transcription in the indicated direction (see labels below each lane). The RNAI transcript is generated from the plasmid DNA replication origin and serves as an internal control. Two units of RNA polymerase holoenzyme (NEB) were used per reaction. Where present, CRP was used at concentrations of 0, 0.5, 1, 2, and 4 μM. Schematic representations of each promoter region derivative are shown below the gel images and are labelled as in prior figures. (**b**) CRP regulation of bidirectional promoters *in vivo*. Promoter sequences, with CRP binding sites in different positions, were fused to *lacZ* in each possible orientation to measure *acrA* or *acrR* transcription. Derivatives of constructs with -35 element mutations that reduce transcription in the *acrA* direction are labelled ‘*acrA* down’. The effect of CRP is seen by comparing expression levels in wild type (open bars) and Δ*crp* (speckled bars) for *acrA*. For *acrR*, the equivalent bars are solid and striped, respectively. Data are the mean of three replicates and error bars show standard deviation. Schematics show organization of the wild-type DNA fragments. The *P-*value was calculated as described in Fig. 2, where *** <.001.

Unlike SoxS and MarA, activators functioning by recruitment can only interact with RNA polymerase when already bound to DNA [9]. To understand the impact at bidirectional promoters, we made two P*acrA*/*acrR* derivatives (schematic in Fig. 3a and Supplementary Table S2). The variants contain an ectopic binding site for the CRP protein. The CRP site was centred either 60.5, or 40.5, base pairs upstream of the *acrA* TSS. These are locations from which CRP is known to activate transcription [2]. Consistent with this, in both cases, *acrA* mRNA production increased upon addition of CRP (Fig. 3a, lanes 1–5 and 11–15). Simultaneously, transcription of the divergent *acrR* mRNA was blocked (lanes 2–5 and 12–15). To understand whether repression was due to CRP binding alone, or a consequence of RNA polymerase interference, we introduced the ‘*acrA* down’ -35 element mutation. This was only possible when CRP bound at position -60.5; the CRP site centred at -40.5 overlaps the -35 hexamer. As expected, the change rendered CRP unable to activate *acrA*. Simultaneously, repression of *acrR*, likely due to RNA polymerase interference, was lost (lanes 6–10).

To further investigate the P*acrA*/*acrR* derivatives, we used promoter::*lacZ* fusions (Fig. 3b). For variants, with the CRP site at position -60.5 or -40.5, *acrAB* transcription decreased when CRP was deleted (compare open and speckled bars, wild-type constructs). Conversely, transcription of *acrR* (solid bars, wild type) increased in cells lacking CRP (striped bars, wild type). The effects were more pronounced when CRP bound position -40.5, consistent with our *in vitro* transcription assays. Experiments with the ‘*acrA* down’ promoter, having an altered -35 element and a CRP site at -60.5, showed an unexpected increase in *acrR* transcription (Fig. 3b, solid bar for ‘*acrA* down’). We speculate that this may be due to better translation, since no similar effect was seen *in vitro* (Fig. 3a, lanes 6–10). Importantly, and despite this overall increase, deletion of CRP had no impact (Fig. 3b, compare solid and striped bars for ‘*acrA* down’). We conclude that activators, whether functioning by recruitment or pre-recruitment, similarly impact bidirectional promoters by generating RNA polymerase interference.

### Binding of MerR family activator SoxR to the P*soxS*/*soxR* bidirectional promoter

**Figure 4.**
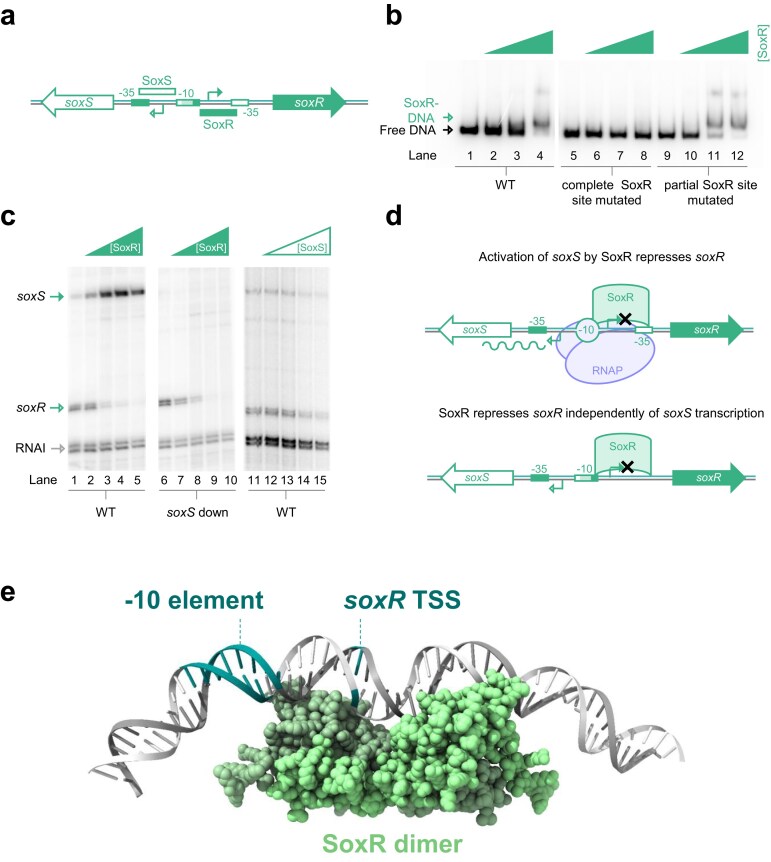
The MerR family activator SoxR directly represses divergent RNA production at the bidirectional promoter P*soxS*/*soxR*. (**a**) Organization of P*soxS*/*soxR*. The schematic shows organization of the bidirectional promoter P*soxS*/*soxR*. Genes (not to scale) are shown as block arrows and TSSs as bent arrows. Promoter elements for forward and reverse transcription are shown as filled and open boxes, respectively. Lighter shading indicates -10 element DNA used for both forward and reverse transcription. Binding sites for transcription factors are shown as elongated boxes and are labelled. (**b**) Binding of purified SoxR protein to P*soxS/soxR*. The panel shows results of electrophoretic mobility shift assays with either the wild-type promoter region or derivatives carrying mutations in two putative SoxR sites. The complete SoxR site is labelled in panel (a) and mutation abolishes SoxR binding. The partial SoxR site, consisting of only one half site, is located between the shared -10 hexamer and the -35 element for transcription in the direction of *soxR*. Mutation of this partial binding site does not alter SoxR binding. Concentrations of SoxR used were 0, 1, 2, or 4 μM. (**c**) SoxR activates *soxS* transcription, and directly represses *soxR* transcription, from P*soxS*/*soxR*. Transcripts generated from the *soxRS* promoter by RNA polymerase holoenzyme in the presence or absence of purified SoxS or SoxR protein. Two units of RNA polymerase holoenzyme (NEB) were used per reaction. Concentrations of SoxR were 0, 0.05, 0.1, 0.2, 0.4 μM. Concentrations of SoxS were 0, 0.375, 0.75, 1.5, 3 μM. The RNAI transcript is derived from the plasmid replication origin and serves as an internal control.(**d**) Model for simultaneous activation and repression by SoxR. (**e**) Structural model of SoxR bound to P*soxS*/*soxR*. The -10 element, used for bidirectional transcription, and the *soxR* TSS are highlighted.

As noted above, MerR type regulators stimulate transcription by kinking the DNA between promoter -10 and -35 elements [13]. To understand consequences for divergent transcription, we turned our attention to P*soxS/soxR* (Fig. 4a). The *soxR* gene encodes a MerR family transcription factor known to stimulate transcription of *soxS* [37]. As a starting point, we sought to understand the number and position of SoxR sites in the *soxS*/*soxR* regulatory region. Previously, Seo and co-workers predicted two such sites. While one prediction matches the complete consensus, the other has only half of the motif needed to bind SoxR [38]. In EMSAs, P*soxS/soxR* DNA shifted primarily into a single complex with SoxR (Fig. 4b, lanes 1–4). Mutation of the complete SoxR site abolished all binding (Fig. 4b, lanes 5–8). Conversely, altering the partial SoxR motif had no effect (Fig. 4b, lanes 9–12). We conclude that SoxR binds in one location only, to activate *soxS* transcription, as shown in Fig. 4a.

### SoxR can activate transcription of one divergent RNA while directly repressing production of the other

For transcription in the direction of *soxS*, core promoter element separation is a sub-optimal 19 bp. This is not the case for initiation of *soxR* mRNA production. Hence, basal transcription in the latter direction is more frequent (Fig. 4c, lane 1). Upon addition of SoxR, transcription in the *soxS* direction is substantially activated. Simultaneously, production of the *soxR* transcript is blocked (lanes 2–5). We repeated the assay using the ‘*soxS* down’ mutant. In this scenario, the absence of a functional -35 element, for *soxS* transcription, renders SoxR unable to activate. However, repression of the divergent *soxR* mRNA was still apparent (Fig. 4c, lanes 6–10). We conclude that MerR family regulators, such as SoxR, oppositely regulate divergent mRNA production, independently of RNA polymerase competition, at bidirectional promoters (Fig. 4d). Presumably, this occurs because regulator binding, within the shared promoter region (Fig. 4a) restricts RNA polymerase access to a single orientation. Consistent with this, structural analysis of the SoxR:P*soxS/soxR* complex, using AlphaFold 3.0, indicates that SoxR occludes the TSS, and part of the -10 element, for transcription of *soxR* [39] (Fig. 4e). Note that we were unable to monitor transcription from P*soxS/soxR* using *lacZ* fusions because activity in both directions was too low. In contrast to SoxR, SoxS caused modest repression of both transcripts (Fig. 4c, lanes 11–15).

### Divergent transcription impacts gene expression noise in bacterial populations

Our observations show that basal transcription, from the tested bidirectional promoters, does not create competition between RNA polymerase molecules. Hence, loss of transcription in one direction has little impact on the divergent RNA (Fig. 1). Conversely, if transcription in one direction substantially increases, production of the opposing transcript is reduced (Figs 2 –4). With these two situations in mind, we wondered whether ensemble experiments may hide important complexity. Specifically, in populations of bacteria, transcriptional noise (cell-to-cell variation) differs for basally expressed, activated, and repressed genes [19, 40, 41]. Hence, if transcription in one orientation is constrained, due to increased production of the opposing RNA, what are the consequences for noise?

**Figure 5.**
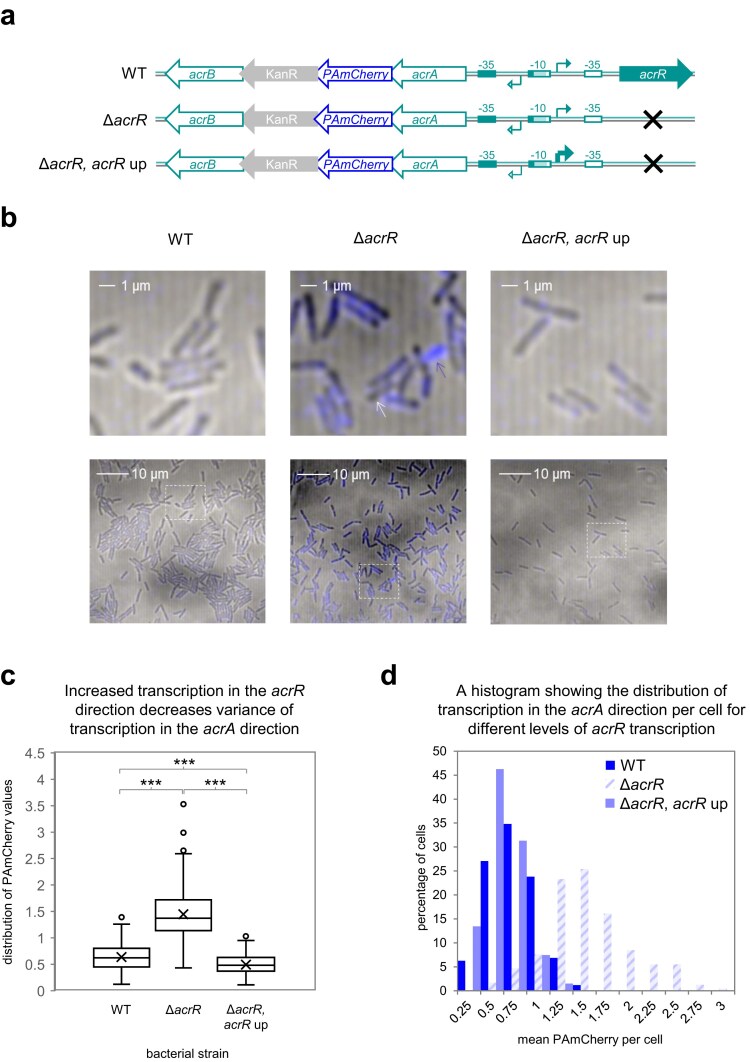
Competition between RNA polymerase molecules at P*acrA*/*acrR* impacts population-level gene expression noise. (**a**) Schematic representation of the *acrAB*/*acrR* locus in different strains. The *E. coli* genome was modified to encode PAmCherry between *acrA* and *acrB* (top). A derivative of this strain was made lacking *acrR* (middle) and this strain was then altered further by improving the -35 element for transcription in the direction of *acrR*. The cross indicates the position of the genetic scar left after deleting *acrR*. (**b**) Representative confocal microscopy images of each bacterial strain. The smaller field of view is the boxed area from the larger field of view for each strain. Cells marked with a blue or white arrow highlight the extremes of PAmCherry expression levels observed for the Δ*acrR* strain.(**c**) Distribution of fluorescence in cells measured by confocal microscopy. The ‘x’ symbol marks the mean fluorescence value per strain. Boxes indicate the lower and upper quartiles, separated by a line indicating the median. Whiskers indicate the largest and smallest data points within 1.5 times the interquartile range. Outliers are shown as individual data points. A two-tailed Student’s *t*-test (assuming unequal variance) was used to calculate *P*. (**d**) Fluorescence intensity as a measure of PAmCherry expression in each strain, shown as a histogram.

To understand links between RNA polymerase interference and transcriptional noise, we focused on the P*acrA*/*acrR* locus. First, the gene encoding PAmCherry was inserted downstream of chromosomal *acrA*. Hence, transcription in the *acrA* direction leads to fluorescence. Next, so that we could measure basal transcription, we removed *acrR*. Recall that AcrR represses transcription in both directions (Fig. 2b). Last, we introduced the ‘*acrR* up’ -35 element mutation to create RNA polymerase interference. The different genetic scenarios are illustrated in Fig. 5a. Cultures were grown until log phase and PAmCherry fluorescence, in individual cells, was measured by confocal microscopy (Fig. 5b). Visual inspection of the data indicates increased fluorescence when *acrR* is deleted (compare left and central panels). Furthermore, the population appears more variable in the latter situation; some cells have high (blue arrow, centre top) and others very low (white arrow, centre top) levels of fluorescence. Increasing transcription in the *acrR* direction (right panel) reduced fluorescence. Visual inspection suggests this population is more homogeneous. The complete data are presented as a box plot in Fig. 5c and a histogram in Fig. 5d. The distribution of fluorescence values was significantly different for each strain. Furthermore, the range of values detected was broadest in the absence of both *acrR* and the ‘*acrR* up’ mutation. To quantify transcriptional noise, we calculated the Fano factor for each strain. This widely used indicator, sometimes referred to as ‘noise strength’, describes the relationship between all values in a distribution (e.g. fluosecence per cell) and the population mean [40, 41]. A larger Fano factor indicates greater noise. For wild-type cells, the Fano factor was 0.10 and this increased to 0.16 upon deletion of *acrR*. Consistent with reduced noise, populations with the ‘*acrR* up’ mutation had a much smaller Fano factor of 0.07. We conclude that interference, between RNA polymerase molecules, constrains both gene expression and noise at the population level.

### Competition at PacrR/acrA increases sensitivity to antibiotics

AcrAB-TolC is a well-characterized efflux pump that removes antibiotics from bacterial cells [34]. In a final set of experiments, we sought to understand the impact of divergent transcription on antibiotic resistance phenotypes. Wild-type cells, and strains with the Δ*acrR* or Δ*acrR*, ‘*acrR* up’ genotypes (as in Fig. 5a, but without the PAmCherry insertion) were grown in increasing concentrations of antibiotic and minimum inhibitory concentrations (MICs) were determined (Table 1). Deletion of the *acrR* gene had no effect on sensitivity to chloramphenicol or novobiocin, consistent with previous studies [42]. Introduction of the ‘*acrR* up’ mutation decreased the MIC for all three antibiotics, suggesting decreased levels of AcrAB-TolC due to increased RNA polymerase competition.

**Table 1.** Changes in MICs due to altered RNA polymerase competition

MIC (μg/ml)^a^			
Strain	Chloramphenicol	Tetracycline	Novobiocin
Wild type	5	0.5	512
Δ*acrR*	5	0.25	512
Δ*acrR, acrR* up	1.25	0.125	64

a MICs were done in duplicate and MICs were identical between duplicates.

## Discussion

Bidirectional promoters are recently discovered, found throughout the prokaryotes, and drive production of divergent RNAs [16, 17]. Since the transcripts produced are frequently mRNAs, we sought to understand bidirectional promoter control. We show that all such promoters tested here exist close to an activity threshold, above which production of one transcript negatively impacts synthesis of the divergent RNA (Fig. 1). Consequently, activators stimulating transcription in one direction tend to inhibit production of the opposing message (Figs 2 –4). In most cases, such repression occurs because RNA polymerase molecules cannot simultaneously engage bidirectional promoters in both orientations. Consistent with this model, if activators are allowed to bind the promoter, but cannot activate, divergent transcription proceeds uninhibited (Figs 2 and 3). This also demonstrates that transcription factor binding does not hinder RNA polymerase transit; the regulator is displaced. Speculatively, at some bidirectional promoters, such eviction of transcription factors may ‘reset’ the promoter to a base regulatory state.

While the above model applies to activators that function by RNA polymerase recruitment or pre-recruitment (Figs 2 and 3) not all regulators work in this way. For example, proteins in the MerR family deform DNA between the promoter -10 and -35 elements to ensure their correct alignment [13, 37]. This mechanism of activation prevents synthesis of the divergent transcript directly. Thus, even if binding of the regulator is uncoupled from transcription activation, synthesis of the opposing RNA cannot occur (Fig. 4). Most likely, this is because regulator binding masks the site of divergent transcription initiation. In the case of the P*soxS*/*soxR* promoter, SoxR activates transcription in the direction of *soxS* and simultaneously prevents its own expression. This contrasts with the situation at P*acrA*/*acrR*. Here, activation in the *acrA* direction, particularly at the levels induced by MarA, still offers an opportunity for *acrR* expression. Such nuances are likely to have important, and yet to be discovered, implications for prokaryotic gene control.

A surprising aspect of bidirectional promoter regulation is the impact on gene expression noise that, in the example studied here, is greater when transcription in one direction does not impinge on the other. Experimentally, this can be observed at P*acrA*/*acrR* when the AcrR repressor is absent; average *acrAB* expression and cell-to-cell variability increase. Conversely, increased *acrR* transcription restricts *acrAB* mRNA production and variability between cells (Fig. 5b). This resembles the degree of variation when AcrR is present. Hence, at least for this promoter, low-level transcription is intrinsically less noisy. Indeed, although unaware of bidirectional promoters, a prior report reached similar conclusions [40]. While cell-to-cell variability is detectable using microscopy (Fig. 5) only batch effects can be observed in MIC assays (Table 1). Even so, it is likely that derepression of P*acrA*/*acrR* creates variable populations. Cell-to-cell differences in antibiotic efflux are likely to result and could be a useful bet-hedging strategy. Given the thousands of bidirectional promoters controlling mRNA production in *E. coli*, and other prokaryotes, our findings provide a framework to understand this newly appreciated level of gene control.

## Supplementary Material

gkag028_Supplemental_Files

## Data Availability

Full gel images are available in Supplementary Fig. S4. Data used to generated graphs, and from individual replicates, are available on request.
