# Supplementary material for: Bidirectional promoters in Escherichia coli: regulatory rules and implications for gene expression noise
Source: Nucleic Acids Res. 2026 Jan 22;54(3):gkag028. doi: 10.1093/nar/gkag028 (PMC12825309; doi:10.1093/nar/gkag028)
Supplement: gkag028_Supplemental_Files [file gkag028_supplemental_files.zip › 130126160349_Supplementray_tables_and_references_130126.docx]

**SUPPLEMENTARY FIGURE LEGENDS**

**Figure S1:** The schematic shows the most common bidirectional promoter arrangements (1). In this situation, divergent transcription start sites (TSSs, shown as arrows) are separated by ~10 bp, 18 bp or 23 bp and rely on a shared -10 region. This provides sufficient symmetry for the region to be used by RNA polymerase in either orientation. This 18 bp spacing is most common because it results in the base at position -18, with respect to each TSS, being a thymine. This facilitates interactions between RNA polymerase and the DNA backbone and is sufficient for transcription to occur in the absence of other promoter elements. Note that small spacing deviations are possible because TSSs are not always 7 bp downstream of the -10 element.

**Figure S2:** Binding of SoxS protein to P*acrA/acrR* with/without the “*acrA* down” mutation. EMSA with radiolabelled promoter DNA and purified SoxS protein. Concentrations of SoxS protein were 0, 0.5, 1, 2 and 4 μM.

**Figure S3:** The gel image shows results of *in vitro* transcription assays for each bidirectional promoter region and derivatives with the -35 element “down” mutation. Either 0.5, 1 or 2 units of RNA polymerase holoenzyme (NEB) were used per reaction. The RNAI transcript is generated from plasmid DNA replication origin and serves as an internal control.

**Figure S4:** Uncropped gel images. Boxes indicate regions used in figures.

**SUPPLEMENTARY DATA LEGEND**

**Supplementary Data S1:** Raw data set presented in Figure 5.

**Table S1: Strains and plasmids**

**Name Description Source**

*Bacterial Strains*

JCB387 Δ*nirB* Δ*lac* (2)

*acrA*-PAmCherry JCB387 with PAmCherry insertion between *acrA* and This work *acrB*, KanR.

Δ*acrR* *acrA*-PAmCherry derivative lacking *acrR* This work

Δ*acrR*, *acrR* up Δ*acrR* derivative having the *acrR* up promoter mutation This work

*Plasmids*

pRW50 Used to make promoter::*lacZ* fusions. Encodes TetR. (3) Low copy number.

pSR Encodes AmpR, derived from pBR322. Features a (4) cloning site upstream of a λ*oop* terminator. Used as a template for *in vitro* transcription assays.

pLSR A derivative of pSR with λoop terminators upstream and (5) downstream of the MCS.

pDOC-C Gene Doctoring vector for genome engineering (6)

pJ203-SoxS Constitutively expresses SoxS, CamR. This work

pET28a Protein expression vector with T7lac promoter Novagen

**References**

1. Warman,E.A., Forrest,D., Guest,T., Haycocks,J.J.R.J., Wade,J.T. and Grainger,D.C. (2021) Widespread divergent transcription from bacterial and archaeal promoters is a consequence of DNA-sequence symmetry. *Nat Microbiol*, 6, 746–756.

2. Page,L., Griffiths,L. and Cole,J.A. (1990) Different physiological roles of two independent pathways for nitrite reduction to ammonia by enteric bacteria. *Arch Microbiol*, 154, 349–354.

3. Lodge,J., Fear,J., Busby,S., Gunasekaran,P. and Kamini,N.R. (1992) Broad host range plasmids carrying the *Escherichia coli* lactose and galactose operons. *FEMS Microbiol Lett*, 95, 271–276.

4. Kolb,A., Kotlarz,D., Kusano,S. and Ishihama,A. (1995) Selectivity of the Escherichia coli RNA polymerase Eσ38 for overlapping promoters and ability to support CRP activation. *Nucleic Acids Res*, 23, 819–826.

5. Samir El-Robh,M. and W Busby,S.J. (2002) The Escherichia coli cAMP receptor protein bound at a single target can activate transcription initiation at divergent promoters : a systematic study that exploits new promoter probe plasmids. *Biochem. J*, 368, 835–843.

6. Lee,D.J., Bingle,L.E., Heurlier,K., Pallen,M.J., Penn,C.W., Busby,S.J. and Hobman,J.L. (2009) Gene doctoring: A method for recombineering in laboratory and pathogenic Escherichia coli strains. *BMC Microbiol*, 9.

**Table S2:** DNA sequences of bidirectional promoters.

**Name Sequence^1^**

*Naturally occurring promoters*

*acrA/acrR* **ctgcg**tttatattatcgtcg**tg**c**tatggt**acatac**a**ttcacaaatgtatgtaaa

gacgcaaatataatagca**g**cacga**taccat**gtatgtaagtgtttacat**acattt**

*acrR* up **TtgACA**ttatattatcgtcg**tg**c**tatggt**acatac**a**ttcacaaatgtatgtaaa

AacTGTaatataatagca**g**cacga**taccat**gtatgtaagtgtttacat**acattt**

*acrA* down **ctgcgt**ttatattatcgtcg**tg**c**tatggt**acatac**a**ttcacaaatgtaACATTT

gacgcaaatataatagca**g**cacga**taccat**gtatgtaagtgtttacatTGTAAA

*soxS/soxR* **ttcgtt**aattcatctgttggggag**tataat**tcctcaa**g**ttaacttgaggtaaa

aagcaattaagta**g**acaacccc**tcatat**taaggagttcaattgaac**tccatt**t

*soxR* up **ttGACA**aattcatctgttggggag**tataat**tcctcaa**g**ttaacttgaggtaaa

aaCTGTttaagta**g**acaacccc**tcatat**taaggagttcaattgaac**tccatt**t

*soxS* down **ttcgtt**aattcatctgttggggag**tataat**tcctcaa**g**ttaacttgaCCATTT

aagcaattaagta**g**acaacccc**tcatat**taaggagttcaattgaactGGTAAA

*bdcA/bdcR* **ttgatt**tattatgtaacatgcat**tacaaa**actgtttt**a**actttctgtcaa

aactaaataatac**a**ttgtacg**taatgt**tttgacaaaattgaaag**acagtt**

*bdcR up* **ttgaCA**tattatgtaacatgcat**tacaaa**actgtttt**a**actttctgtcaa

aactGTataatac**a**ttgtacg**taatgt**tttgacaaaattgaaag**acagtt**

*bdcA down* **ttgatt**tattatgtaacatgcat**tacaaa**actgtttt**a**actttcACAGTT

aactaaataatac**a**ttgtacg**taatgt**tttgacaaaattgaaagTGTCAA

*bioA/bioB* **ttgtaa**accaaattgaaaagatt**taggtt**tacaagt**c**tacaccgaattaacaacaaa

aaacaAacatttggtttaacttttctaaat**c**caaatgt**tcagat**gtggcttaattgttgtt**ttttgt**

*bioB up* **ttgACa**accaaattgaaaagatt**taggtt**tacaagt**c**tacaccgaattaacaacaaa

aaacaaacTGttggtttaacttttctaaat**c**caaatgt**tcagat**gtggcttaattgttgtt**ttttgt**

*bioA down* **ttgtaa**accaaattgaaaagatt**taggtt**tacaagt**c**tacaccgaattaacaacaaT

TTTGTaacatttggtttaacttttctaaat**c**caaatgt**tcagat**gtggcttaattgttgttAAAACA

*fepD/entS* **ttatcg**atcttatttggata**tg**t**tagcat**gtgcagcctaagaataggtaatagctag

aat**a**aaccta**tacaat**c**gt**acacgtcggattcttatcca

*entS up* **ttatcg**atcttatttggata**tg**t**tagcat**gtgcagc**c**taagaTGTCAAaatagctag

aat**a**aaccta**tacaat**c**gt**acacgtcggattct**ACAGTT**

*fepD down* AATAGCatcttatttggata**tg**t**tagcat**gtgcagc**c**taagaataggtTTATCGtag

aat**a**aaccta**tacaat**c**gt**acacgtcggattcttatcca

*Semi-synthetic promoters P*acrA/acrR *CRP site mutations*

CRP -60.5 **ttgctg**cgtttatattatcgtcgtgc**tatggt**acatac**a**ttcacaaatgtatgtaaatctaac

gcctgtaaattAGTgaacatatTCACAgaaaaaccaaaaacgacgcaaatataatagca**g**cacga**taccat**gtatgtaagtgtttacat**acattt**agattgcggacatttaaTCActtgtataAGT

GTctttttggttt

CRP -60.5 **ttgctg**cgtttatattatcgtcgtgc**tatggt**acatac**a**ttcacaaatgtaACATTTtctaac

*acrA* down gcctgtaaattAGTgaacatatTCACAgaaaaaccaaaaacgacgcaaatataatagca**g**cac

ga**taccat**gtatgtaagtgtttacatTGTAAAagattgcggacatttaaTCActtgtataAGTGTCtttttggttt

CRP -40.5 **ttgctg**cgtttatattatcgtcgtgc**tatggt**acatac**a**ttcacaaatgtatgtTGTGAtaac

gcTCACaaattcacgaacatatggcacgaaaaaccaaaaacgacgcaaatataatagca**g**cac

ga**taccat**gtatgtaagtgtttacat**aca**ACACTattgcgAGTGtttaagtgcttgtataccgtgctttttggttt

^1^ Transcription start sites obtained by cappable-seq are underlined. Predicted promoter elements are in bold. Mutated bases are shown in capitals.
