# Supplementary figures and images for: Bidirectional promoters in Escherichia coli: regulatory rules and implications for gene expression noise
Source: Nucleic Acids Res. 2026 Jan 22;54(3):gkag028. doi: 10.1093/nar/gkag028 (PMC12825309; doi:10.1093/nar/gkag028)

**Figure S1**

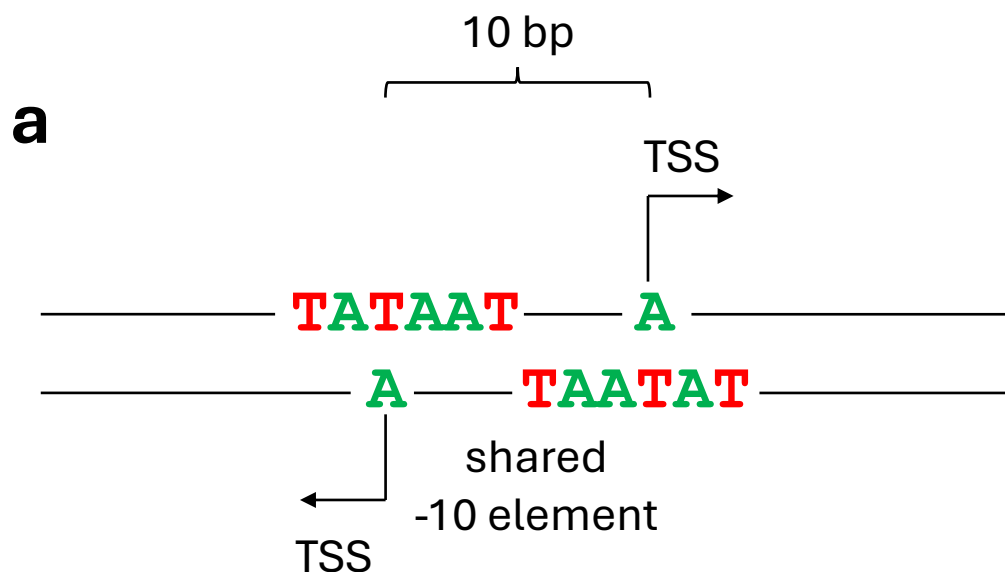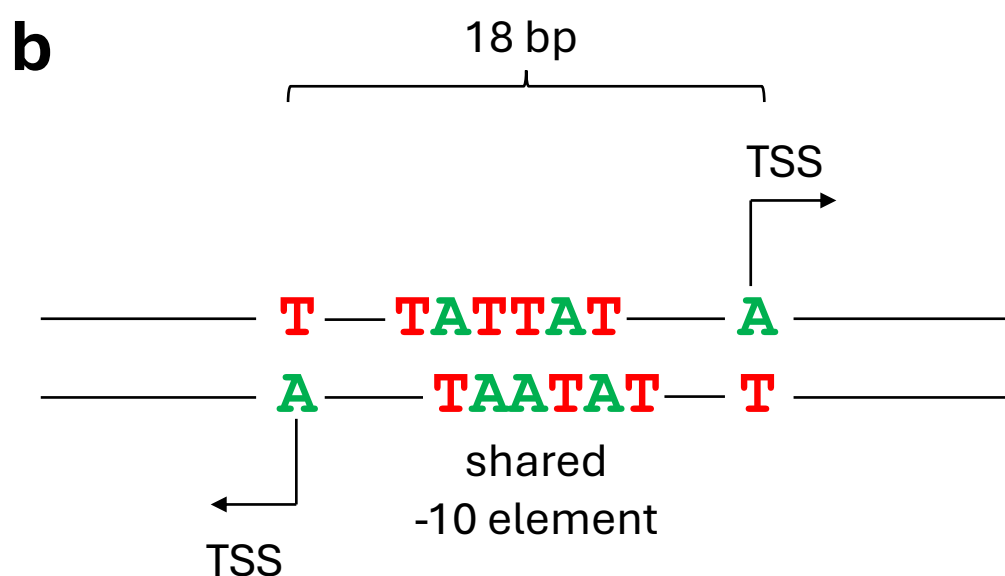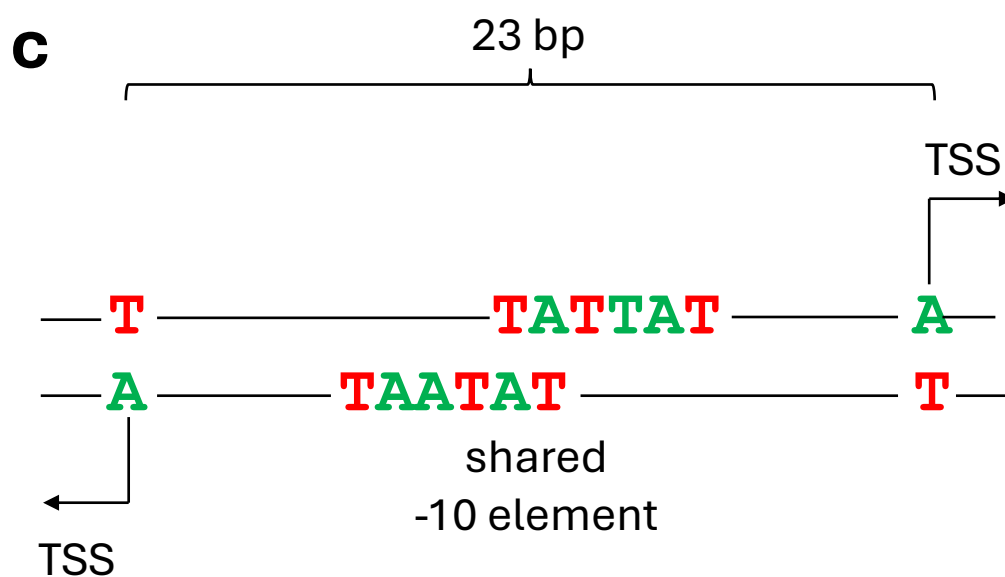

Figure S2

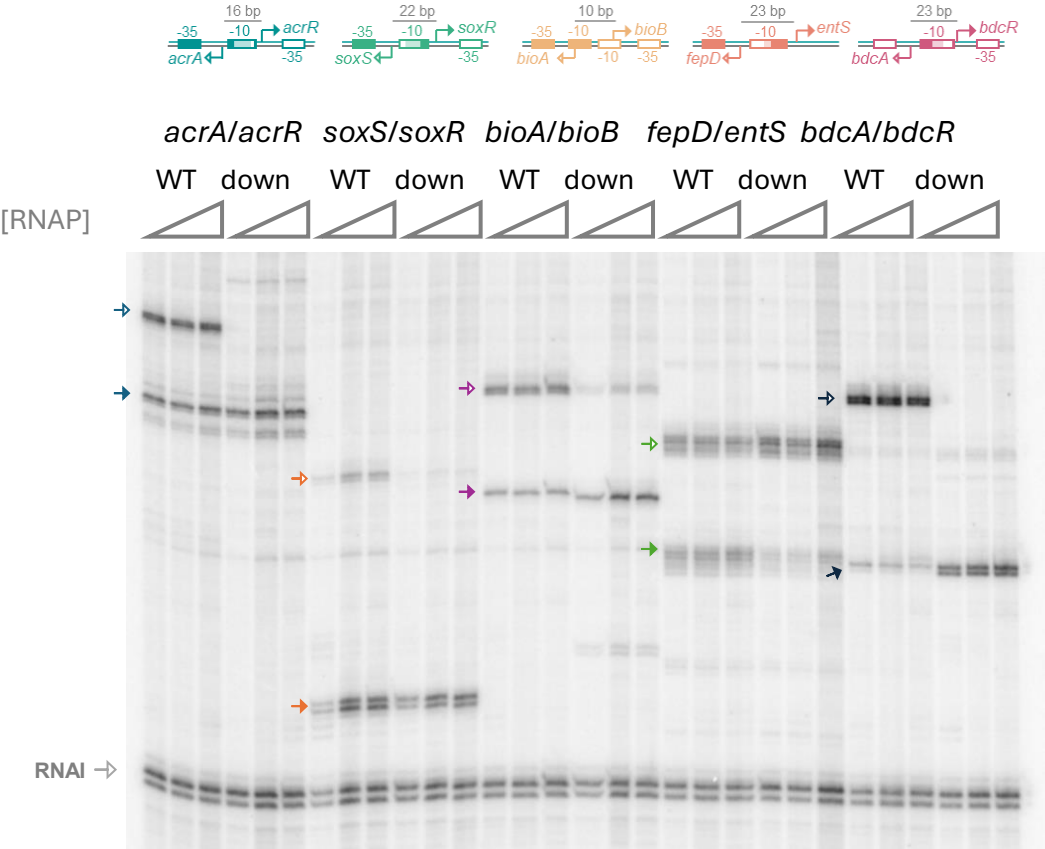

Figure S3

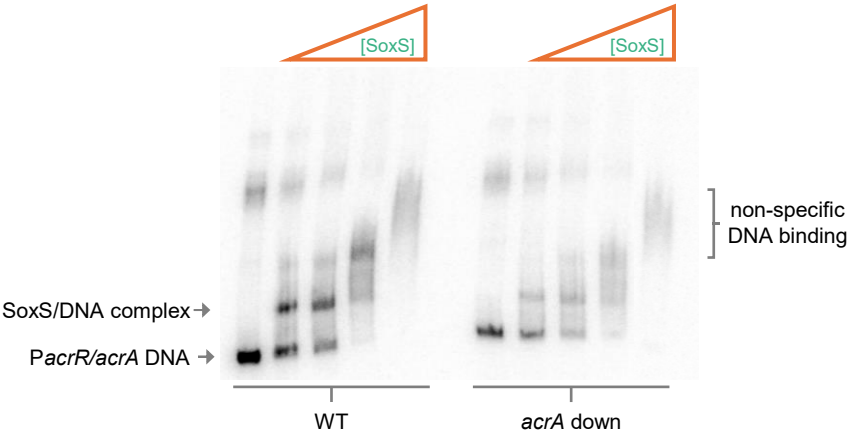

Figure 1

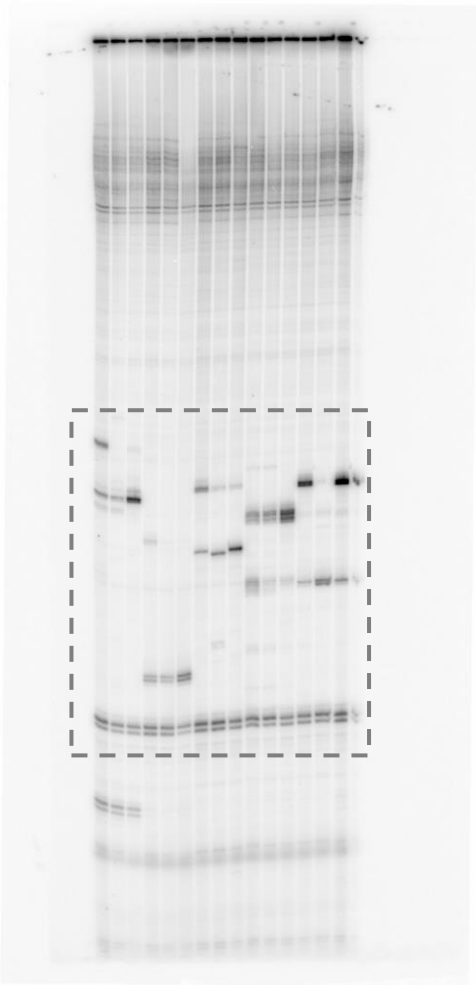

Figure S4

Figure 2

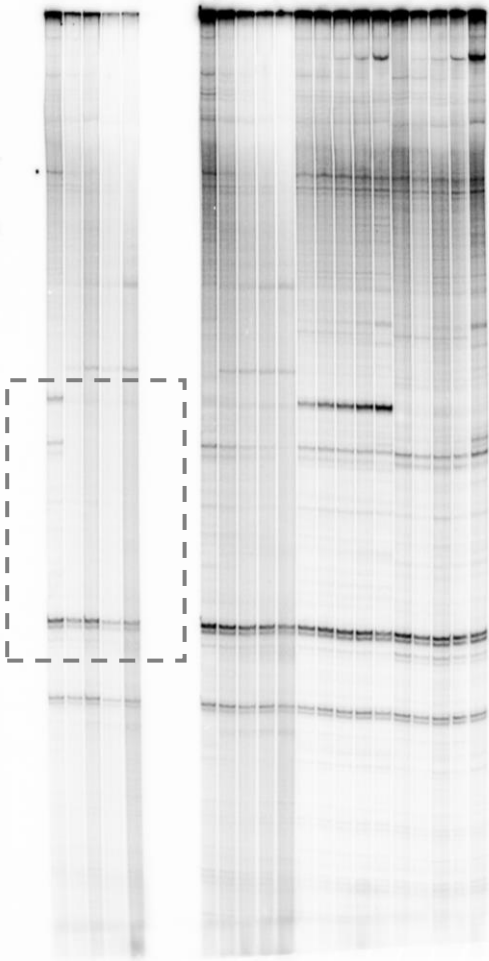

Figure 2

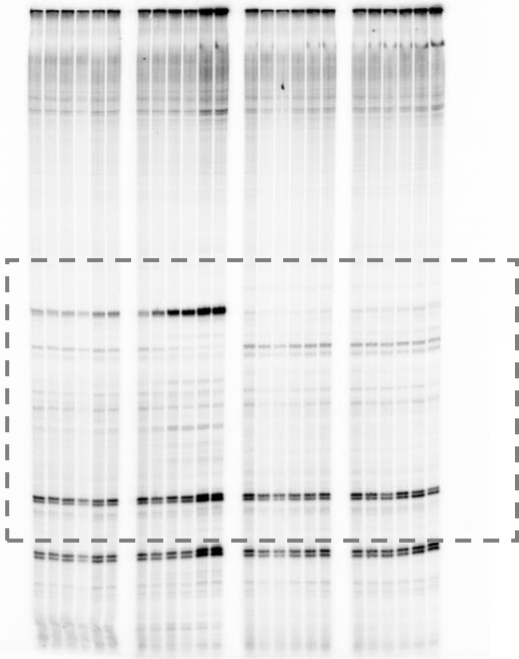

Figure 3

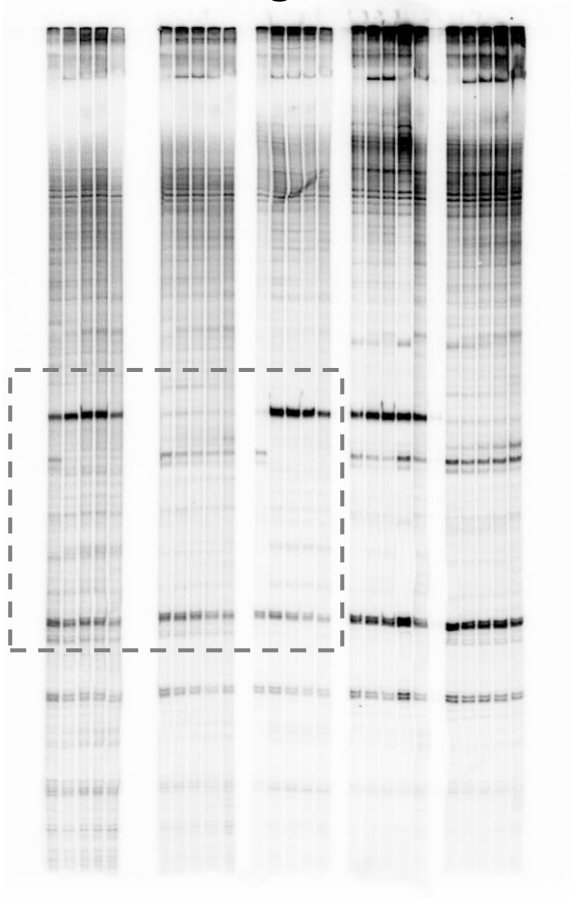

Figure 4

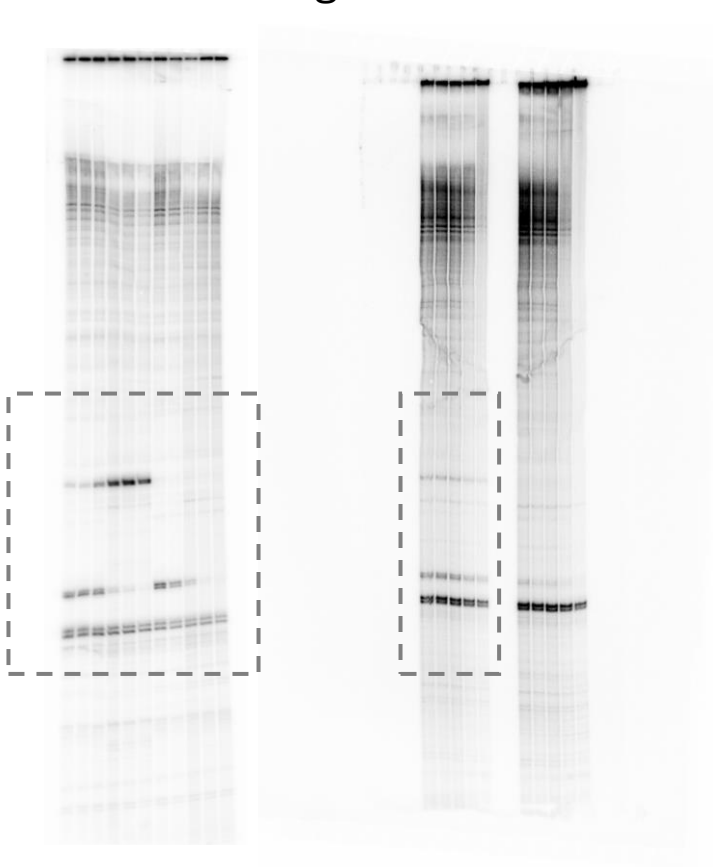

Figure S2

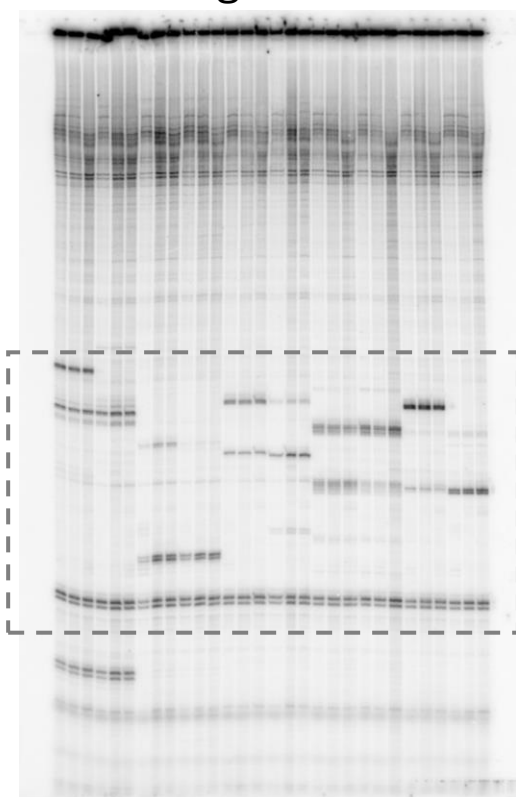

Figure S3

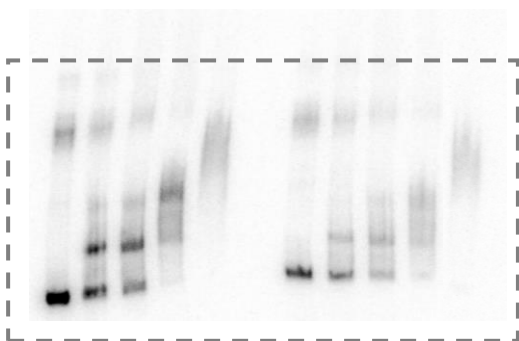

Supplement: gkag028_Supplemental_Files [file gkag028_supplemental_files.zip › Supplementary Figures 25112025.pdf]
